# Acceptance of medical drone technology and its determinant factors among public and healthcare personnel in a Malaysian urban environment: knowledge, attitude, and perception

**DOI:** 10.3389/fpubh.2023.1199234

**Published:** 2023-11-17

**Authors:** Muhammad Za’im Sahul Hameed, Rosdiadee Nordin, Aniza Ismail, Muhammad Aidiel Zulkifley, Aina Suraya Helmy Sham, Raja Zahratul Azma Raja Sabudin, Mohamed Afiq Hidayat Zailani, Ismail Mohd Saiboon, Zaleha Abdullah Mahdy

**Affiliations:** ^1^Department of Community Health, Faculty of Medicine, Universiti Kebangsaan Malaysia, Kuala Lumpur, Malaysia; ^2^Department of Electrical, Electronic and Systems Engineering, Faculty of Engineering and Built Environment, Universiti Kebangsaan Malaysia, Bangi, Malaysia; ^3^Department of Pathology, Faculty of Medicine, Universiti Kebangsaan Malaysia, Kuala Lumpur, Malaysia; ^4^Department of Emergency Medicine, Faculty of Medicine, Universiti Kebangsaan Malaysia, Kuala Lumpur, Malaysia; ^5^Department of Obstetrics and Gynecology, Faculty of Medicine, Universiti Kebangsaan Malaysia, Kuala Lumpur, Malaysia

**Keywords:** medical delivery drone, drone acceptance, medical practitioners, public community, KAP model

## Abstract

**Introduction:**

Unmanned aerial vehicles (UAVs) are used for commercial, medical, public safety, and scientific research purposes in various countries.

**Methods:**

This study aimed to explore the acceptance of medical delivery drones among medical practitioners as well as the public community in Malaysia using a knowledge, attitude, and perception (KAP) model and statistical analysis to decrease uncertainty. Bivariate and multivariate analyses of the results were performed in SPSS.

**Results:**

A total of 639 respondents took part in the survey, of which 557 complete responses were finally analyzed. The results showed that the overall acceptance rate for medical delivery drones was positive. The acceptance rate was significantly correlated with knowledge, attitude, and perception scores but not with sociodemographic factors.

**Discussion:**

Raising awareness and educating the medical as well as public communities regarding the potential role and benefits of drones are therefore important in garnering support for drone usage for medical purposes.

## Introduction

Medical drone delivery was first established in Rwanda and Ghana by zipline, where delivery of life-saving blood, blood specimens, and medical supplies is performed between medical facilities and hospitals (1). Such methods are utilized more in rural areas due to the lack of infrastructure for land transport. Many researchers have since then used Zipline as the primary reference for implementing medical drone delivery services. The use of medical drone delivery services has, however, raised several issues, including the invasion of privacy and concerns about safety (2).

Drone technology advancement in terms of industrial processes and communication and networking technologies has led to their widespread use in civil, business, and social applications (3, 4). The recent COVID-19 pandemic has forced many countries to present innovative measures, which will also widen the use of drones in civil, commercial, and social applications, especially for the transportation of medicine and medical care where conventional methods are too inefficient or slow for emergency situations (5).

There is a broad application for drones in medicine, ranging from the delivery of medicines (6), emergency transport of blood products in maternal healthcare (7), and even combating the COVID-19 pandemic (8). In Africa, drones are already saving lives by delivering blood packets to remote villages (9). The use of drones to transport blood products in the event of a clinical emergency has been compared with ambulances and shown to significantly shorten transport time (10). Four studies have shown that drones were useful in out-of-hospital cardiac arrest (11). Three reports documented that drones can assist in the search for lost or injured people (12). Drones have even been found to improve healthcare delivery through faster response times, reductions in transport costs, enhanced access to medical products and services in remote or underserved environments (13), and measures of air pollution (14). These benefits act as factors that may be conducive to public acceptance of drones. The usage of drones in Papua New Guinea, Asia, and Africa was well accepted, according to a review by Poljak and Sterbenc in 2020 (15).

In Malaysia, the three most common causes of maternal mortality were associated medical conditions, postpartum hemorrhage (PPH), and obstetric pulmonary embolism (16). Approximately two-thirds of public clinics in Malaysia are located in rural areas (5). There is a need for a rapid blood delivery system in the management of obstetric emergencies in order to handle PPH. Drones or UAVs could potentially prevent maternal mortality in obstetric emergencies, mainly in rural areas, especially in Sabah and Sarawak, where there are abundant mountainous terrains, beaches, and tropical rainforests (7). The drones’ rapid response time, expected reduction in transportation costs, and improved medical product or service delivery to remote areas such as the interior of Sabah give them a high potential to be a holistic solution for improved healthcare in Malaysia (7). Long-term evolution (LTE/4G) networks in Malaysia, combined with Reference Signals Received Power (RSRP) and Reference Signal Received Quality (RSRQ) modeling, have been found to provide a reliable communication link for UAVs at a distance of up to 1 km and an elevation angle of up to 85 degrees (17).

The earliest Malaysian study regarding the adoption of unmanned aerial technology (UAT) was conducted by Chamata from Curtin University in 2016, revealing that 66.5% of the public community agreed that UAT is beneficial (18). In our study, we set out to measure knowledge, attitude, and perception (KAP) scores regarding medical drone delivery services among medical practitioners and members of the public community in an urban area of Malaysia and to relate these determinants, as well as sociodemographic background, to their acceptance of such drone services.

## Materials and methods

A questionnaire survey was developed following the KAP model. The selection of the domains and formulation of the questions were largely on Chamata et al. (18) and Aydin (19). Initially, a pilot survey was collected from 46 respondents, both medical practitioners and public community members, to test the reliability of the survey questions. The number of questions in each of the three knowledge, attitude, and perception (KAP) domains and the corresponding Cronbach alpha values are shown in Table 1. Table 2 shows the operative definitions of the sociodemographic variables. The list of questions in the questionnaire is accessible at http://tinyurl.com/3m77jzrb.

**Table 1:** Item reliability.

Domain	Number of questions	Cronbach Alpha
Knowledge	6	0.80
Attitude	6	0.80
Perception	6	0.80

**Table 2:** Operative definition of sociodemographic variables.

Variable	Operative definition
Age	Age of respondent in years
Gender	Biological sex of respondent
Education	Highest stage of learning completed by the respondent
High education	Tertiary education
Drone user	Whether or not the respondent had used a drone previously

A sample size calculation was performed based on population data and previous studies involving surveys of perspectives and attitudes regarding drone deliveries. The population in Selangor was estimated to be 6.56 million (20). The number of medical practitioners in this region was the highest in Malaysia at 9,483 (21). Sample size calculation was performed using power and sample size calculation (PS), producing a minimum sample size of *n* = 369 for the public community and *n* = 231 for medical practitioners at a confidence level of 95%.

The survey was distributed online via various platforms, such as WhatsApp, Instagram, Facebook, and e-mail. Only respondents who lived in the Klang Valley and above 18 years of age were allowed to proceed with the survey.

## Results

A total of 639 responses were received, of which 256 were medical practitioners and 383 were members of the public (community members). Among 256 medical practitioners, 173 (67.7%) were female respondents, while 83 (32.3%) were male respondents, whereas out of 383 respondents from the community, 226 were female respondents, while the remaining 157 were male respondents. However, 82 responses were incomplete; hence, the final number of respondents was 557, of which 224 were medical practitioners and 333 were public community members. The sociodemographic background of the respondents is depicted in Table 3. Tables 4, 5 show the profile of the two big groups of respondents.

**Table 3:** Sociodemographic profile of all respondents.

Variable	Category	*N* (%)
Gender	Male	218 (39.1)
	Female	339 (60.9)
Age	18–29	107 (19.2)
	30–39	183 (32.9)
	40–49	139 (25.0)
	50–59	111 (19.9)
	60 and above	17 (3.1)
Education	Low	20 (3.6)
	High	537 (96.4)
Drone user	Yes	55 (9.9)
	No	502 (90.1)
Medical practitioner	Yes	224 (40.2)
	No	333 (59.8)

**Table 4:** Sociodemographic profile of medical practitioners.

Variable	Category	*N* (%)
Gender	Male	75 (33.5)
	Female	149 (66.5)
Age	18–29	16 (7.1)
	30–39	110 (49.1)
	40–49	55 (24.6)
	50–59	41 (18.3)
	60 and above	2 (0.9)
Education	Bachelor	86 (38.4)
	Masters	113 (50.4)
	PhD	22 (9.8)
	Others	3 (1.3)
Occupation	Consultant	26 (11.6)
	House officer	3 (1.3)
	Medical officer	99 (44.2)
	Senior consultant	23 (10.3)
	Specialist	64 (28.6)
	Others	9 (4.0)
Drone user	Yes	9 (4.0)
	No	215 (96.0)

**Table 5:** Sociodemographic profile of public community members.

Variable	Category	*N* (%)
Gender	Male	143 (42.9)
	Female	190 (57.1)
Age	18–29	91 (27.3)
	30–39	73 (21.9)
	40–49	84 (25.2)
	50–59	70 (21.0)
	60 and above	15 (4.5)
Education	Foundation/matriculation	7 (2.1)
	Diploma	49 (14.7)
	PhD	41 (12.3)
	Baccalaureate	61 (18.3)
	Bachelor	162 (48.6)
	Malaysian Certificate of Education	11 (3.3)
	Others	2 (0.6)
Occupation	Self-employed	27 (8.1)
	Private sector worker	127 (38.1)
	Student	31 (9.3)
	Civil servant	116 (34.8)
	Unemployed	20 (6.0)
	Other	12 (3.6)
Drone user	Yes	46 (13.8)
	No	287 (86.2)

### Acceptance

Overall acceptance was positive, with 82.9% agreeing to drone usage. The acceptance in the community was slightly higher, with a mean Likert scale score of 4.62 and an acceptance rate of 85.0%, while among medical practitioners, it was 4.50 and 79.9%, respectively.

Due to a skewness level of −2.08 and a kurtosis level of 3.37, the data were not normalized, and thus a Mann–Whitney test was more appropriate than a *t*-test. There were no violations of the Mann–Whitney test detected, and medical practitioner and community member groups were independent of each other, while the dependent variable of acceptance was ordinal. The mean rank for medical practitioners’ acceptance was 270.63, while the mean rank for community acceptance was 284.63, with a significance value of 0.123 (*p* > 0.05), demonstrating that there was no significant difference between the two groups in terms of drone acceptance.

Other than knowledge, attitude, and perception, sociodemographic factors were also analyzed to observe how these factors affected the acceptance of medical drone delivery. It was surprising to find that 11 and 9% of drone users from among medical practitioners and public community members, respectively, declined the usage of medical drone delivery compared to non-drone users, which were 5 and 3%, respectively. No public community member from a low educational background rejected the idea of medical drone delivery. In brief, none of the sociodemographic determinants were significantly associated with drone acceptance.

Table 6 shows the results of bivariate chi-square tests on the sociodemographic factors of medical practitioners and their effect on drone acceptance. The low chi-square values show a weak association between sociodemographic factors and drone acceptance. None of the factors were significant (*p* > 0.05), thus demonstrating that sociodemographic factors were unrelated to drone acceptance.

**Table 6:** Bivariate analysis of medical practitioner sociodemographic factors.

Variable	Chi-Square	*p*-value
Gender	5.26	0.07
Age	0.56	0.76
Drone user	0.84	0.66
Education	5.21	0.51

Table 7 shows the results of bivariate chi-square tests on the sociodemographic factors of public community members and their effect on drone acceptance. The low chi-square values show a weak association between sociodemographic factors and drone acceptance. None of the factors were significantly associated (*p* > 0.05), thus demonstrating that sociodemographic factors were unrelated to drone acceptance.

**Table 7:** Bivariate analysis of community members’ sociodemographic factors.

Variable	Chi-square	*p*-value
Gender	2.76	0.252
Age	2.22	0.33
Drone user	3.27	0.20
Education	11.75	0.47

### Knowledge

**Figure 1:**
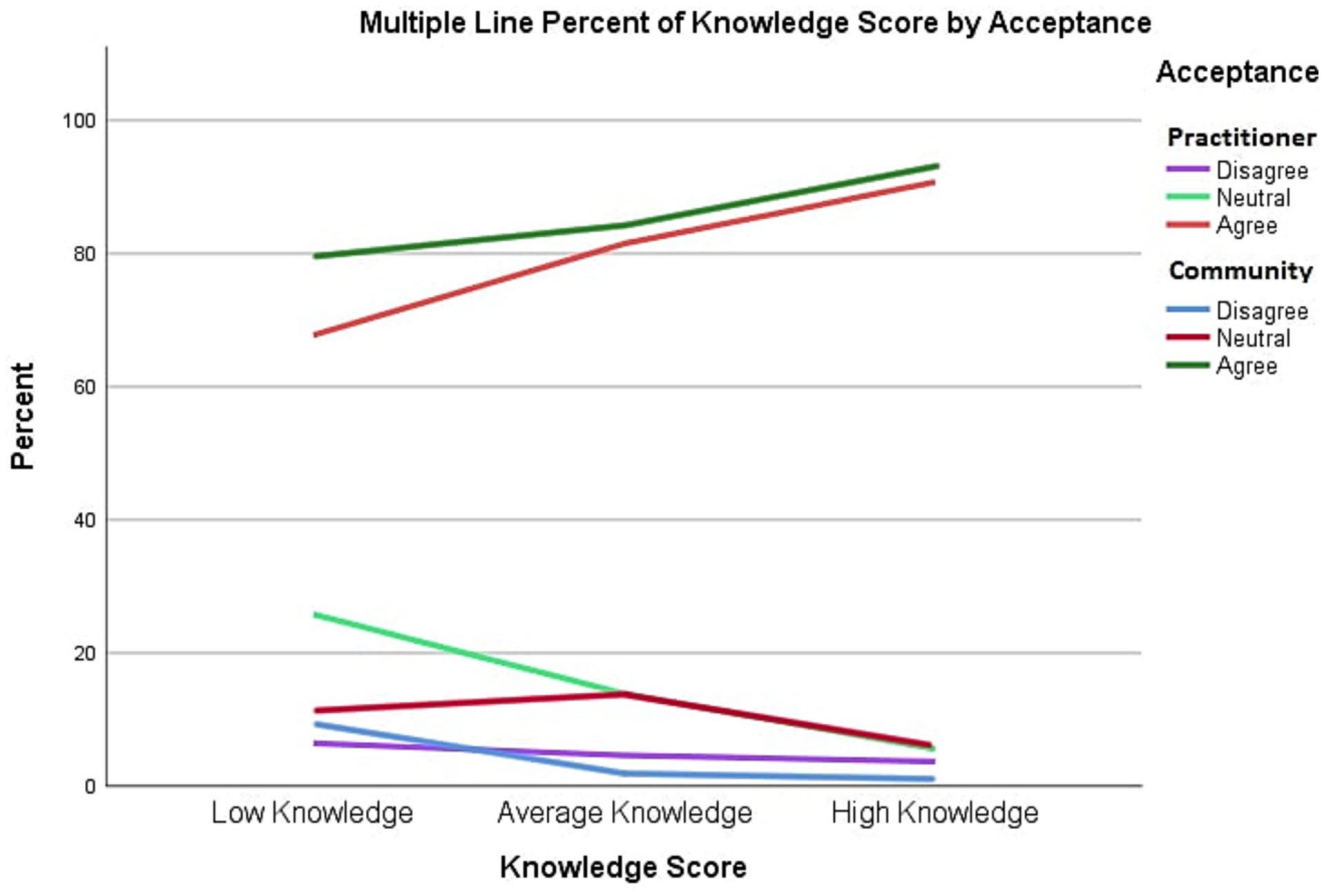
Relationship between acceptance and knowledge scores.

A chi-square test was conducted, showing a significant association between knowledge and acceptance level; *p* = 0.033, *α* = 0.05 for the medical practitioner group and *p* = 0.007 for the public community members group. The results show that high levels of knowledge were associated with a high acceptance level of medical drone delivery (see Figure 1).

**Figure 2:**
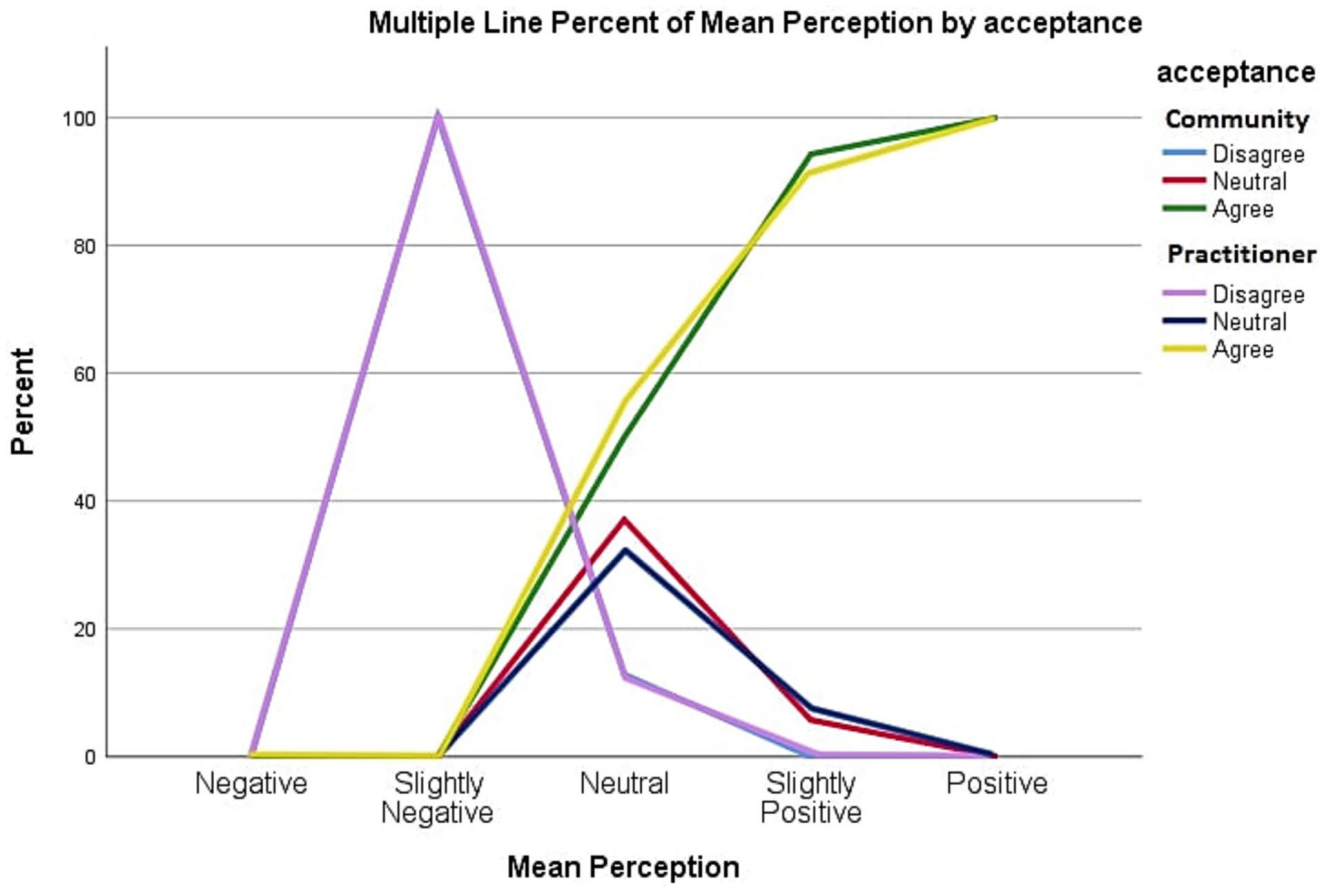
Perception versus acceptance in community members and medical practitioners’ groups.

To normalize the data, we used percentages of acceptance scores to assess the overall acceptance score for each participant. The acceptance score was taken as the response variable. Figure 2 shows the relationship between knowledge scores and drone acceptance among both medical practitioners and public community member groups. Acceptance scores among both public community members and medical practitioners increased as the knowledge score increased. There is a slightly higher percentage of public community members who accept drone usage than medical practitioners. As the knowledge scores increase, the number of those who were neutral or disagreed with drone usage decreases. The percentage of those who were neutral among the public community members and medical practitioners overlaps as their numbers decrease at high knowledge scores.

Table 8 shows the results of bivariate chi-square analysis on KAP scores among public community members and medical practitioners. The high chi-square values coupled with significant value of *p*s (<0.05) show that KAP scores were significantly associated with drone acceptance.

**Table 8:** Bivariate analysis of KAP for medical practitioners and community members.

Variable	Chi-square value	*p*-value
Knowledge		
Medical practitioner	10.48	0.03
Public community	14.21	0.01
Attitude		
Medical practitioner	35.53	<0.05
Public community	162.09	<0.05
Perception		
Medical practitioner	61.34	<0.05
Public community	204.81	<0.05

### Attitude

The mean score of attitude was analyzed to test for significance. The overall mean Likert scale score of attitude was 3.97, which falls under “Slightly Positive” attitude for medical practitioners, while the score for public community members was 4.18. The chi-square test was conducted, demonstrating a significant association between attitude and acceptance level with a value of *p* of <0.001 for both medical practitioners and public community member groups. The results suggest that a positive attitude was significantly associated with acceptance of medical drone delivery.

**Figure 3:**
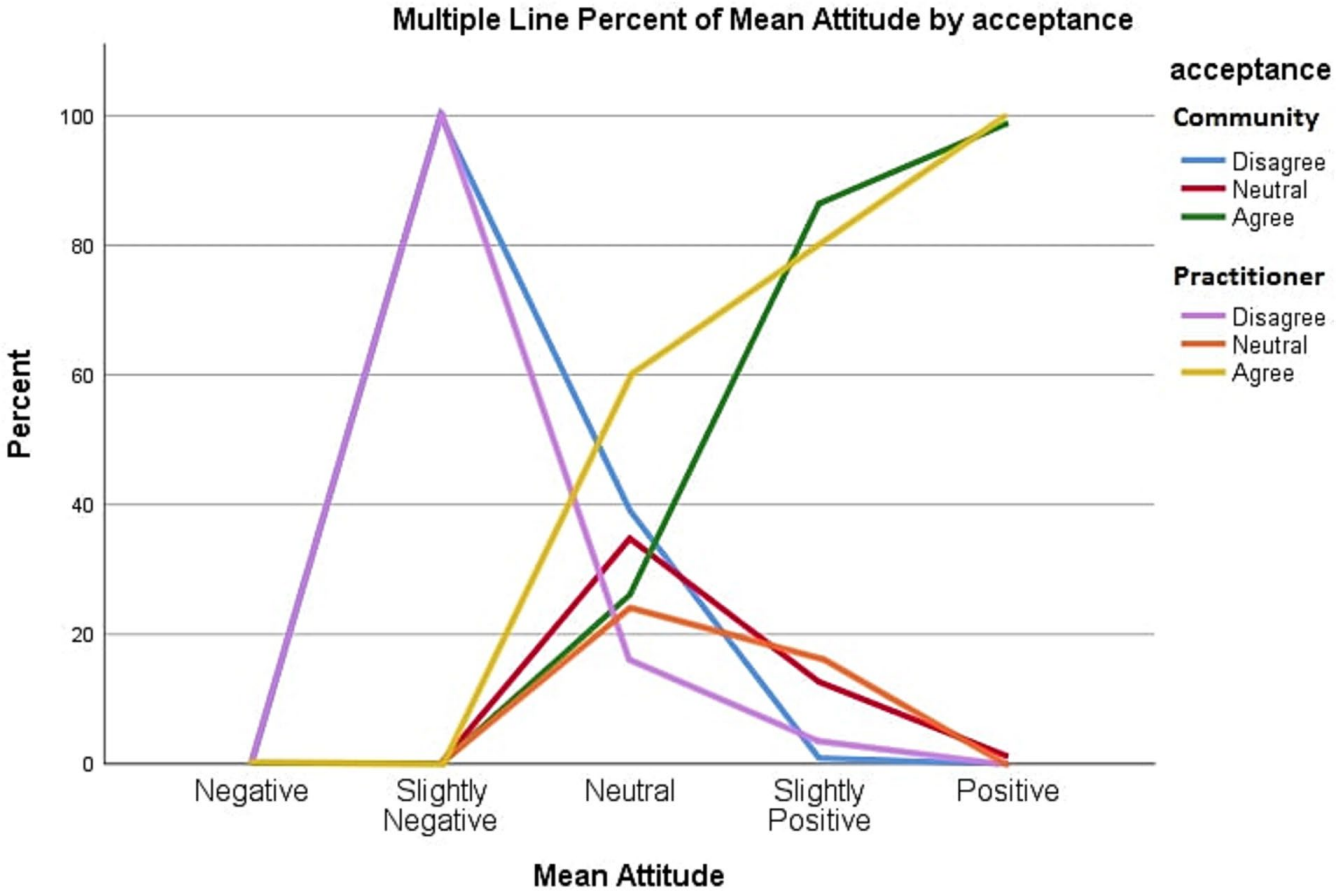
Relationship of percentage of mean attitude against acceptance scores.

Figure 3 shows a multiple-line graph demonstrating the relationship between attitude and acceptance among medical practitioners and community members. As the attitude scores increase, drone acceptance increases in both community members and medical practitioner groups. The increase in acceptance rates in both groups almost overlaps with each other.

### Perception

The same method was used to generate results for perception and the overall mean perception Likert scale scores for medical practitioners and public community members were 3.67 and 3.83, respectively, which also fell under the “Slightly Positive” group. The chi-square test was also conducted, showing a statistically significant association between perception and acceptance of medical drone delivery, with a value of *p* of <0.01 for both medical practitioners and public community member groups. This indicates that positive perception leads to a high acceptance level of medical drone delivery.

The graph in Figure 2 shows the relationship between perception and acceptance among public community members and medical practitioner groups. Acceptance in both groups increases as perception scores increase. The line for disagreement between public community members and medical practitioners’ groups overlaps nearly perfectly.

Table 9 shows the descriptive statistics for the knowledge, attitude, and perception scores of medical practitioners and public community members. Since it is in percentages, the maximum for all groups is 100%. By comparing means, we can see that the knowledge scores of both groups were very similar, but the medical practitioners scored slightly higher (65.48%) than the public community members (65.37%). However, the means of attitude and perception scores were higher for the public community group (83.60 and 76.58%, respectively) compared to the medical practitioners (79.91 and 74.74%, respectively).

**Table 9:** Descriptive statistics for scores among medical practitioners and public community members (%).

Group	Score category	Mean	Standard deviation	Median
Medical practitioner	Knowledge	65.48	24.01	66.67
	Attitude	79.91	9.93	80
	Perception	74.73	10.84	80
Public community	Knowledge	65.37	24.58	66.67
	Attitude	83.60	11.36	80
	Perception	76.58	10.82	80

Repeated measures of ANOVA were conducted to model the acceptance rates among various groups. The results are shown in Table 10. The ANOVA results showed that knowledge, attitude, and perception scores were significant factors for drone acceptance (knowledge *p* = 0.04, perception *p* = 0.00, and attitude *p* = 0.00). Multicollinearity was not detected as variance inflation factors (VIFs) are under 1.12 (VIF < 3.0), with no interaction effects found. Knowledge, attitude, and perception scores were thus examined further.

**Table 10:** Correlation coefficients and their significance toward drone acceptance.

Variable	Significance	Coefficient of determination
Knowledge	<0.01	0.029
Attitude	<0.01	0.254
Perception	<0.01	0.294
Gender	0.14	0.004
Age	0.41	0.001
Education	0.35	0.002
Drone user	0.23	0.003
Medical practitioner	0.16	0.003

The Mann–Whitney test was utilized an over independent *t*-test as there was non-normality of the data. The test showed that low and high knowledge scores differed significantly in drone acceptance with a mean rank of 288.36 and 255.57, respectively (one-tailed exact significance *p* = 0.001). For attitude scores, the Mann–Whitney test revealed that those with high attitude scores differed in drone acceptance in comparison to those with low attitude scores, with mean ranks of 291.51 and 154.83, respectively (one-tailed exact significance *p* = 0.000). Finally, perception scores were tested using the Mann–Whitney test, and it was shown that those with high perception scores were significantly different than those with low perception scores, with mean ranks of 285.92 and 258.39, respectively (one-tailed exact significance *p* = 0.005).

## Discussion

Unmanned aerial vehicles (UAVs) are used for commercial, medical, public safety, and scientific research purposes in various countries. This study aimed to explore the acceptance of medical delivery drones among medical practitioners as well as members of the public community in an urban area in Malaysia, using a knowledge, attitude, and perception (KAP) model and statistical analysis to decrease uncertainty. Bivariate and multivariate analyses of the results were performed in SPSS. A total of 639 respondents took part in the survey, of which 557 complete responses were finally analyzed. The results showed that the overall acceptance rate for medical drones was 82.9%. The acceptance rate was significantly correlated with knowledge, attitude, and perception scores.

Various determinants of drone acceptance have been explored (22). We chose the knowledge, attitude, and perception (KAP) model based on the earlier study conducted by Chamata et al. in 2016, focusing on the humanistic aspect of drone acceptance, and Aydin (19) on public acceptance. With the advent of social media, the knowledge explosion over the past 7 years since the previous study was conducted may have shaped societal awareness and hence modified their attitude and perception of this technology. Progress in drone technology development and its enhanced usage during the recent COVID-19 pandemic could also have colored all three domains of knowledge, attitude, and perception among medical practitioners and public community members. KAP surveys are important because they reveal misconceptions or misunderstandings that may represent obstacles to the activities we would like to implement and potential barriers to behavior change (23).

An important step toward the rapid proliferation of drone delivery is to understand the complex variations in public risk beliefs about the technology (24). Overall, drone acceptance by both public community members and medical practitioners was favorable, with 82.9% in favor of drone usage. Public community members had a higher acceptance rate of 85% in comparison to medical practitioners (79.9%). Other studies have found that the acceptance of drone usage in medicine was often high (5). People were more likely to accept drone usage in rescue operations and medical transportation rather than for parcel delivery or advertising (25).

Regression analysis showed that in both public community members and medical practitioner groups, sociodemographic factors have no significance on drone acceptance. Other studies have shown that female respondents tend to be more critical of drone delivery in general (26, 27). On the other hand, there are also studies reporting that gender has a minor or insignificant influence on drone acceptance (28). Those from urban areas were found to be more accepting of drone usage in comparison to rural dwellers (25). However, that study did not focus on medical drone delivery. A study by Alice Tam found that sociodemographic variables have no strong influence on risk perceptions of drone usage (29). The respondents’ perceptions depended on technology reliability and higher perceived safety with a human pilot on board.

Our study found that age did not have a significant impact on the acceptance of drone usage. Those who were above and below 40 years of age did not demonstrate any significant difference in drone acceptance. Other studies have found that younger respondents were more positive toward drone usage than older ones (30).

Education was also found to not significantly affect the outcome of drone acceptance in our study. In Pakistan, a study found that the young, urbanized, and educated with a steady income and stable lifestyle were more accepting of drone usage (31). A study in Zanzibar found that higher levels of education were linked to higher drone acceptance (32). Whether this could be linked to casual information via high social media exposure among Malaysians is a point to consider.

It is interesting to note that a higher percentage of drone users declined the use of medical drone delivery compared to non-drone users. Hypothetically, this may be attributable to an increased awareness among drone users of potential issues with the drone, such as technological limitations, legislative challenges, and safety and privacy concerns. There may also be a sampling bias, as the sample of drone users in this study may not represent all drone users. On the contrary, non-drone users may be more open to the idea of drone usage for medical delivery because they do not have preconceived notions or experiences that shape their views as a result of a lack of familiarity with the drone. These speculative assumptions require further research, surveys, or interviews to explore the true underlying reasons for this phenomenon.

Conversely, a study in Germany found that possession of drone pilot licenses did not impact the attitude toward drone usage (5). The attitude of the public toward drones may be conditional, as they are more likely to support a service provided by an unmanned aircraft for their own personal benefit (31). It was found in a recent study that the public was more likely to have a positive attitude toward drone usage in search and rescue operations, firefighting, meteorology, emergency response, and disease spread detection than in food delivery, personal recording, or drone racing (19).

Our study revealed that knowledge scores were strongly correlated with drone acceptance. As knowledge scores increased, so did the acceptance of drone usage in both the public community and medical practitioner groups. In 2016, a study found that the general public learned about drones mainly from movies and mainstream news media (33). A study by the Office of the Inspector General of the United States Postal Service in 2017 found that knowledge drives enthusiasm for drone usage (25). High knowledge scores were found to correlate positively with drone acceptance in a study in Germany (30).

Attitude scores were found to be significantly associated with drone acceptance. People’s perception that an innovative service is better than a traditional service might positively affect their adoption of the innovation (34). Higher attitude scores correlated positively with drone acceptance. A 2019 study showed that even though rural societies would benefit more from drone usage, their attitude was similar to that of urban respondents due to their lack of knowledge (19). Yoo et al. reported in 2019 that there were differences in attitudes between urban and rural groups in an online survey. Both groups were concerned with speed, ease of delivery, and performance risk, while the rural group’s concern for environmental friendliness and personal innovation had a significant effect on their attitude toward drone delivery (25).

Using a bivariate analysis in SPSS, this study found that perception scores were strongly correlated with drone acceptance. A study in the UK and Italy found that privacy concerns played a key role in the perception of drones (35). Cultural misconceptions regarding what a drone can do pose a challenge to the rapid adoption of drones (36). In a 2016 study, respondents perceived that drones were beneficial for operations, but budget, manpower, and regulations pose obstacles to the adoption of the technology (37). A recent study in Switzerland demonstrated that the perception of drones depended upon where they were used and what their purpose was (37). According to a nationwide study, public perception of drone usage was influenced by the purpose for which the drones would be employed. Positive public perception of drone usage prevailed in situations where the benefit of drones outweighed the associated risks, such as in search and rescue, disaster response, law enforcement, border surveillance, terrorist monitoring, and crime prevention (38). A study by Reddy in 2016 found that public perception of drones was low for commercial and homeland security applications (33).

The main limitation of this study is that the survey only covers the Klang Valley area, which represents an urban area in Malaysia; none of the respondents were from rural areas. Hence, the study did not consider the opinions of rural dwellers. Nonetheless, the Klang Valley is the largest single urban area in the country; hence, it may be considered representative of urban Malaysia, as we proposed to study an urban population. The final number of responses analyzed was slightly lower than the calculated sample size; hence, the power of our study was slightly lower than the targeted 80%. There may also be non-response bias and sampling bias as a result of the sampling method that was used. Moreover, the questions listed in the knowledge domain were quite brief and may not have strongly reflected the knowledge level. This had to be done to keep the survey short and attractive to respondents. Furthermore, because this is one of the earliest studies on medical drone services in Malaysia, the local literature review was limited.

## Conclusion

In conclusion, medical drone delivery is accepted by both medical practitioners and public community members. Knowledge, attitude, and perception all strongly and positively influenced drone acceptance, while sociodemographic factors including gender, age, drone usage, and education level were not significantly associated with acceptance of drone usage in medicine. Raising awareness and educating the medical and public communities regarding the potential role and benefits of drones are therefore important in preventing objections and garnering support for drone usage for medical purposes in future.

## Data availability statement

The raw data supporting the conclusions of this article will be made available by the authors, without undue reservation.

## Ethics statement

The study involving humans was approved by Kebangsaan Malaysia Research Ethics Committee. The study was conducted in accordance with the local legislation and institutional requirements. The participants provided their written informed consent to participate in this study.

## Author contributions

ZM and RN: conceptualization, writing – review and editing, and funding acquisition. MZa, MZu, ZS, IS, AI, and AS: methodology. MZa’, AI, and AS: formal analysis. ZS, IS, and AS: data curation. MZa’: writing – original draft preparation. RN: supervision and project administration.
